# Predictive Factors for Massive Transfusion in Trauma: A Novel Clinical Score from an Italian Trauma Center and German Trauma Registry

**DOI:** 10.3390/jcm9103235

**Published:** 2020-10-10

**Authors:** Sara Giulia Cornero, Marc Maegele, Rolf Lefering, Claudia Abbati, Shailvi Gupta, Fabrizio Sammartano, Stefania Cimbanassi, Osvaldo Chiara

**Affiliations:** 1 Trauma Team and General Surgery ASST Niguarda Milano, Department of Pathophysiology and Transplantation, University of Milano, 20125 Milano, Italy; saragiuliacornero@gmail.com (S.G.C.); claudia.abbati@unimi.it (C.A.); fabrizio.sammartano@ospedaleniguarda.it (F.S.); stefania.cimbanassi@ospedaleniguarda.it (S.C.); 2 Cologne-Merheim Medical Center, Department for Trauma and Orthopedic Surgery, Institute for Research in Operative Medicine, University Witten/Herdecke, 51109 Köln, Germany; maegelem@kliniken-koeln.de (M.M.); Rorlf.Lefering@uni-wh.de (R.L.); 3 R Adams Cowley Shock Trauma Center, University of Maryland Medical System, Baltimore, MD 21201, USA; shailvi.gupta@gmail.com

**Keywords:** trauma, bleeding, blood transfusion, score

## Abstract

Early management of critical bleeding and coagulopathy can improve patient survival. The aim of our study was to identify independent predictors of critical bleeding and to build a clinical score for early risk stratification. A prospective analysis was performed on a cohort of trauma patients with at least one hypotensive episode during pre-hospital (PH) care or in the Emergency Department (ED). Patients who received massive transfusion (MT+) (≥4 blood units during the first hour) were compared to those who did not (MT−). Hemodynamics, Glagow Coma Score (GCS), diagnostics and blood tests were evaluated. Using multivariate analysis, we created and validated a predictive score for MT+ patients. The predictive score was validated on a matched cohort of patients of the German Trauma Registry TR-DGU. One hundred thirty-nine patients were included. Independent predictors of MT+ included a prehospital (PH) GCS of 3, PH administration of tranexamic acid, hypotension and tachycardia upon admission, coagulopathy and injuries with significant bleeding such as limb amputation, hemoperitoneum, pelvic fracture, massive hemothorax. The derived predictive score revealed an area under the curve (AUC) of 0.854. Massive transfusion is essential to damage control resuscitation. Altered GCS, unstable hemodynamics, coagulopathy and bleeding injuries can allow early identification of patients at risk for critical hemorrhage.

## 1. Introduction

Critical bleeding secondary to trauma occurs rapidly after injury [1,2] and remains a common cause of potentially avoidable deaths [3]. The Advanced Trauma Life Support (ATLS) class three and four shock represent life threatening conditions due to loss of 30% or more of circulating blood [4]. Hemorrhage after trauma is often associated with the progressive onset of coagulopathy due to a combination of factors such as hemodilution, consumption of coagulation factors, hypothermia, acidosis and liberation of cellular mediators with activation of the neuroendocrine axis. Trauma-induced coagulopathy (TIC) [5] is associated with a greater need for transfusions, an increase in complications and mortality [6].

Early recognition and aggressive treatment of bleeding through combined strategies of damage control resuscitation (DCR) [7], damage control surgery (DCS) [8] and endovascular hemostatic procedures, may save lives. DCR which includes the use of massive transfusion (MT) protocols aims to correct circulating volume while preserving coagulation capability, all being associated with a reduction in TIC magnitude and less inappropriate use of blood products [8,9]. This approach should be considered in all patients with severe traumatic injuries who show severe hemorrhage, altered physiology, metabolic imbalance and thereby reduced physiologic reserve [9]. As a recent study demonstrated patient mortality increases by 5% for every minute that passes following MT protocol activation while awaiting initial transfusion [1].

The objective of this study was to identify a set of variables easily obtainable during the early phase of care for the severely injured patient. Such variables will allow a rapid risk stratification for ongoing critical bleeding and for timely activation of DCR principles with the use of MT.

## 2. Materials and Methods

This research was conducted through collaboration between the University of Milano Trauma Center at Niguarda Hospital (Milan, Italy) and the Institute for Research in Operative Medicine (IFOM) Cologne-Merheim Medical Center (CMMC) at the Campus Cologne-Merheim of the University of Witten/Herdecke (Köln, Germany). The study was approved by the Ethical Committees of the Niguarda Hospital (534-102018, 26 October 2018) and the University of Witten/Herdecke as well as by the Boards of the Institute for Research in Operative Medicine (IFOM), University of Witten/Herdecke, Cologne-Merheim Medical Center (CMMC) and the Review Board of the TR-DGU. TR-DGU data are exclusively based on routinely available data, and patients consented to the use of their data. No patient details like names, addresses, or dates of birth were collected. Furthermore, the scientific dataset was additionally blinded for date of trauma and hospital identifier. A separate ethics vote was thus not mandatory.

### 2.1. Development of the Scoring System

#### 2.1.1. Inclusion and Exclusion Criteria

Data of trauma patients admitted to the Niguarda Hospital were prospectively collected between February 2016 and December 2018. Inclusion criteria were: (i.) age ≥ 18 years, (ii.) Hospital Trauma Team activation by the emergency call center, and (iii.) at least one episode of systolic blood pressure (SBP) < 90 mmHg during PH setting or upon arrival in the ED. Exclusion criteria were: (i.) isolated brain (TBI) or spinal cord injury to avoid potential hemodynamic bias, (ii.) confirmed pregnancy, (iii.) third degree burns in any body region, (iv.) secondary referral from another hospital, (v.) death within the first 30 min of ED arrival and (vi.) ED arrival > 120 min after trauma. Demographics, Injury Severity Score (ISS), type of trauma (blunt or penetrating), PH and ED vital parameters (GCS, SBP, HR, Revised Trauma Score, RTS), PH tranexamic acid (TXA) administration, positive abdominal extended focused abdominal sonography for trauma (E-FAST), pelvic fracture, hemothorax, limb amputation, blood lactate and base excess (BE) levels on admission and standard coagulation parameters (e.g., PT, PTT, platelets count) were evaluated.

#### 2.1.2. Treatment Protocol

Hemodynamically unstable patients as defined by the Advanced Trauma Life Support (ATLS^®^) criteria of classes III or IV at the scene were treated with one gram bolus of TXA by the PH personnel. In non-responder patients, (SBP persistently < 90 mmHg notwithstanding repeated 250 mL crystalloid bolus infusions), hypotesizing a critical bleeding, MT (≥4 blood units within the first 60 min from ED admittance) is foreseen. Massive transfusion protocol was administered as follow: - two 0-negative packed red blood cell promptly available on ED, are transfused, followed by cross-matched packed red blood cells (PRBCs)—fresh frozen plasma (FFP)—platelets (PLT) at PRBCs:FFP:PLT 1:1:1 ratio. In our hospital the average volume of a PRBC is 250–300 mL per bag, obtained by removing 200–250 mL of plasma from 450–500 mL of whole blood.

In addition, cryoprecipitate 1 unit/10 kg body weight if fibrinogen < 2 gr/L in order to correct the consumption of coagulation factors and TXA 1 gr infusion over 8 h, to avoid or counteract the fibrinolysis are administered.

Crystalloid infusion was reduced to a minimum and colloids avoided during both PH and in-hospital care. This protocol remained unchanged throughout the study period. The source of bleeding was addressed and identified in the ED through chest X-ray, pelvis X-ray and E-FAST. A contrast-enhanced whole-body CT-scan (WBCT) was carried out only after hemodynamic stabilization. All DCS techniques were applied if indicated, including damage control laparotomy, extra- peritoneal packing, emergency thoracotomy, limb amputation, external long bone and pelvis fixation. Angioembolization was performed if WBCT after surgery indicated persistent bleeding. Resuscitative endovascular balloon occlusion of the aorta (REBOA) was not available during the study period.

In patients with normalizing SBP over 90 mm Hg after initial crystalloids infusion, MT protocol was not activated and blood transfusions were eventually driven when required by hemoglobin levels.

#### 2.1.3. Statistical Analysis

Patients were divided into those who had critical bleeding and those who did not. MT was defined as 4 or more blood units during the first hour of admission to the ED (MT+) [9]. Those patients with absent signs of critical bleeding and no massive transfusion protocol initiated were defined as MT−. Statistical analysis was performed using the IBM SPSS Statistics version 21 Software. The continuous variables were expressed as mean with standard deviations (M ± SD) and median and compared using the Student’s *t* test. The categorical variables were compared by chi-square test. A *p*-value < 0.05 was considered statistically significant. For each potential predictive variable for MT a logistic model was generated to obtain an odds ratio (OR) with relative 95% confidence intervals to investigate relationships between variables and MT. Clinically relevant variables were entered into multivariable logistic regression models to identify independent predictors of MT and to derive a score for early clinical decision-making.

### 2.2. Validation of the Scoring System

The derived clinical score was validated on a dataset extracted from the German TraumaRegister^®^ (TR-DGU) which yielded a comparable cohort of trauma patients to the one used for score development.

The TraumaRegister DGU^®^ (TR-DGU) of the German Trauma Society (Deutsche Gesellschaft für Unfallchirurgie, DGU^®^) was founded in 1993. The aim of this multi-center database is a de-identified and standardised documentation of severely injured patients. Data are collected prospectively in four consecutive time phases from the site of the accident until discharge from hospital: (A) Pre-hospital phase, (B) Emergency department and initial surgery, (C) Intensive care unit and (D) Discharge. The documentation includes detailed information on patient characteristics, injury pattern, comorbidities, pre- and in-hospital management, course on the intensive care unit, relevant laboratory findings including data on transfusion and outcome of each individual. The inclusion criterion is admission to hospital via the emergency room with subsequent ICU/ICM care or reach the hospital with vital signs and die before admission to ICU. The infrastructure for documentation, data management, and data analysis is provided by AUC—Academy for Trauma Surgery (AUC-Akademie der Unfallchirurgie GmbH), a company affiliated to the German Trauma Society. The scientific leadership is provided by the Committee on Emergency Medicine, Intensive Care and Trauma Management (Sektion NIS) of the German Trauma Society. The participating hospitals submitted their de-identified data into a central database via a web-based application. Scientific data analysis was approved according to a peer review procedure laid down in the publication guideline of TraumaRegister DGU^®^. Participation in TR-DGU is voluntary. For hospitals associated with TraumaNetzwerk DGU^®^, however, the entry of at least a basic data set is obligatory for reasons of quality assurance.

The validation dataset included trauma patients that had been treated in TR-DGU-affiliated German trauma centers during the years 2015–2016 who fulfilled the same inclusion/exclusion criteria previously described. The derived score obtained by multivariable analysis from the Italian cohort was tested on the German population and modified, verifying the frequency of single variables in patients who received MT, in order to obtain the best fitting area (AUC) under the receiving operating curve (ROC). This modified score validated on TR-DGU was finally re-applied on the initial Italian population with AUC analysis to obtain the definitive “Milano Score”.

Briefly, to develop the Milano score the following steps were performed:

- comparison of MT+ and MT− patients with descriptive statistical analysis on an Italian trauma population prospectively included;
- identification with univariate analysis of variables significantly associated with MT+;
- identification with multivariate analysis of independent predictors of MT+ in the Italian dataset and definition of the score;
- validation and adjustment of the score on the TR-DGU^®^ German population, retrospectively evaluated;
- re-application of the final score on the original Italian population.

Manuscript preparation was guided by the STROBE statement for the reporting of cohort studies in epidemiology [10].

## 3. Results

Between February 2016 and December 2018, a total of 2131 trauma patients were admitted to the Emergency Department of the University of Milano’s Level 1 Niguarda Trauma Center (Milan, Italy). Out of these, 139 patients (6.5%) met the inclusion criteria and were thus eligible for the present analysis. Twenty-four patients (17.3%) had GCS = 3. Ninety-one per cent sustained a blunt mechanism of trauma and more than half of the patients (56.8%) were victims of traffic accidents. Seventy-eight patients were MT+ and 61 patients MT−. The characteristics of the study population are presented in Table 1. Of the 139 patients, 33 (23.7%) patients died: 24.2% of deaths occurred in the ED, 21.2% in the operating theatre and the remaining 54.5% in the Intensive Care Unit (ICU). MT+ patients were more severely injured as reflected by significantly higher ISS scores and displayed a higher mortality rate. PH-HR, PH-GCS, PH administration of TXA, SBP-ED and HR-ED, were significantly different in the two groups. The same variables in addition to a penetrating mechanism of injury were all found to be significantly associated with MT with an OR > 2 at univariate analysis. Of the 106 surviving patients, 97 (91.5%) were admitted to the ICU with an average length of stay of 15 ± 18 days, 12 ± 11 days in the MT− group versus 19 ± 24 days (*p* < 0.05) in the MT+ group.

Laboratory variables and clinical findings at initial diagnostic work-up and their association with MT are shown in Table 2. Lactate, base excess (BE), platelet counts, INR and PTT were all significantly different between the two groups. All these variables, but lactate, were found to be significantly associated with MT at univariate analysis. Altered INR and PTT upon ED admission displayed higher odds ratios. Pelvic fracture, hemothorax and limb amputation all correlated with critical bleeding, with limb amputation being the most significant variable. On the other hand, unilateral femur fracture and positive abdominal E-FAST were not associated with the need of MT.

The independent predictors of MT+ are listed in Table 3. Ten variables were identified as independent predictors of MT. The first nine were used to empirically define the predictive score: a score of 1 was assigned to the first eight variables, while a score of 2 was given to PTT ≥ 40 s due to higher odds ratios calculated. The maximum score value was 10. Limb amputation had a very high OR and alone predicted MT. Notably, positive E-FAST became a significant predictor of MT at multivariable analysis, with an elevated OR.

The data extraction process applied to the TR-DGU yielded dataset of 905 patients, 29.8% of which received a MT, with all variables available for score calculation (Table 4). Cross tabulations were created to evaluate the relation of single variables of selected German population with MT and ROC curves were created testing how to obtain the higher AUC. Limb amputation was no longer considered separately in the score, penetrating trauma was not confirmed as predictor of MT and the PTT ratio was assigned with a score of 1.

**Figure 1:**
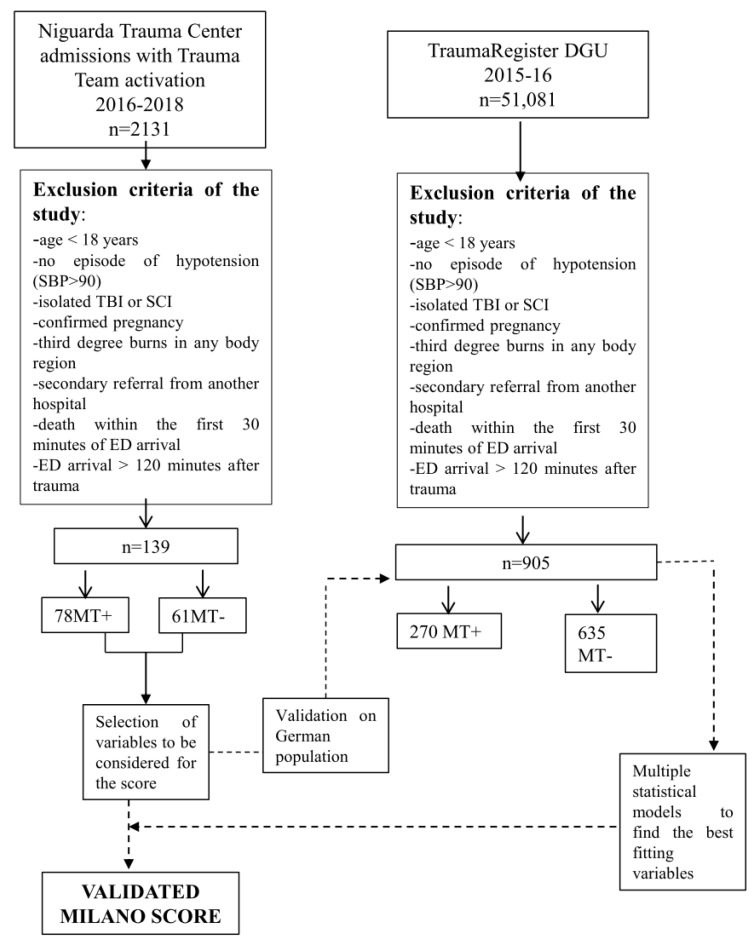
Cohort characteristics and process of validation of the Milano score. The figure describes the steps followed to obtain the validated Milano score. ED: emergency department; MT: massive transfusion; SCI: spinal cord injury; TBI: traumatic brain injury.

The steps followed to obtain the Milano score, and the characteristics of both cohorts of patients are depicted in Figure 1.

**Figure 2:**
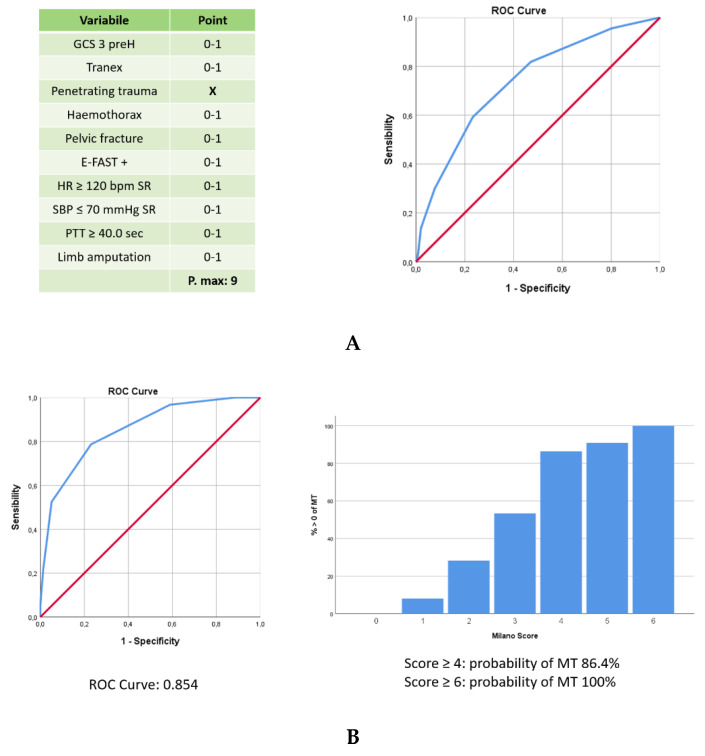
Variables considered in a multivariate analysis to elaborate the predictive score for MT. Figure 2 describes the ten variables used to elaborate the predictive score for MT, their ROC curves in the study populations, and the probability of MT of the validated “Milano score”. Particularly, Figure 2A lists the variables selected on a multivariate analysis tested on german population, identifying a score with AUC of 0.738. Figure 2B represents the validated score (Milano score), applied on Milano population, with an AUC of 0.854. The probability of MT according with different score values is depicted in the histogram. (**A**) Application of the predictive score of MT on the trauma population selected from DGU; (**B**) The predictive score of MT applicated on the Italian population.

The modified score (Figure 2) with a maximum value of 9 obtained an area under the ROC of 0.738. This final score, called Milano score, was finally re-applicated on the original Italian dataset obtaining a larger AUC, with a value of 0.854. Probability of MT was 85.4% with a score ≥ 4 and 100% with a score ≥ 6.

## 4. Discussion

In the present study the following steps were performed for the development of the Milano Score: we first compared MT+ and MT− patients with descriptive statistical analysis on an Italian trauma population prospectively included, afterwhich we identified with univariate analysis variables significantly associated with MT+. Using these variables, we used multivariable analysis in the Italian dataset to define a predictive MT+ score. We then validated and adjusted the score based on the TR-DGU German population, retrospectively evaluated. We then were able to re-apply the final score on the original Italian population.

Our results demonstrate that the need of MT can be predicted by a combination of clinical and laboratory parameters in addition to E-FAST and X-rays, easily obtained during the initial evaluation of the trauma patient. A patient who is comatose and tachycardic on the field, who becomes hypotensive during transportation or in the ED, with laboratory signs of early coagulopathy and evidence of a bleeding injury in the chest, abdomen, pelvis or with a limb amputation, has the maximum chance to require a MT protocol for a critical bleeding. Independent predictors of MT were used to create a score which may be useful to the clinician to initiate MT treatment of the patient. This can serve as a process improvement oriented toward early recognition of critical bleeding and immediate blood product availability allows for mortality reduction.

In past years, six scores have become popular for the prediction of MT need, the accuracy and sensitivity of which were variable according to the cases considered: Trauma Associated Severe Hemorrhage (TASH) [11], Prince of Wales [12], Vandromme [13], ABC [14], Schreiber [15], Larson [16] scores. All these studies defined MT as the need of 10 PRBC or more during the first 24 h after admission and the TASH score, directly derived from TR-DGU was the best performing as predictor of MT (AUC 0.88) [17]. A recently developed new score in Japan, the Traumatic Bleeding Severity Score (TBSS) [18] and its modification [19] furtherly improved ability to predict MT (AUC > 0.9) but requires a detailed classification of the pelvic fracture and the number of positive windows with FAST. In general scores that use clinical assessment, laboratory exams, E-FAST, showed a greater ability to discriminate between patients MT+ and MT−.

In the present study, critical bleeding was defined as the need of ≥4 PRBC in the first hour [9], thus identifying with certainty a population of trauma patients affected by severe bleeding. While this definition is not globally utilized, because we were attempting to identify patients who would benefit from MT+ early, we logically used greater than 4 units of PRBCs within an hour as an indication of requiring a significant amount of blood product. Moreover, all the published scores were developed from retrospective analysis of trauma databases, while Milano score was initially derived from consecutively admitted critical bleeding patients prospectively included in the study.

Our data shows that tachycardia and hypotension in the ED, but not in PH settings, predict MT. Trauma is a dynamic condition and many times an initially normotensive patient becomes hypotensive after few minutes, during transportation or at admission in the ED. Tachycardia is the earliest sign of ongoing bleeding and reduced blood pressure may follow in few minutes. Accordingly, the trend over time of hemodynamic parameters, from PH to ED, and not a single value should be considered. Notably, of the 139 patients with at least one episode of hypotension in PH-ED time, only 61 (44%) required a MT. The persistence of an altered hemodynamic status of a patient upon arrival in ED notwithstanding bolus infusions is more alarming than the hypotension recorded on the field. The pre-hospital shock index (SI), easily calculated from the ratio between heart rate and SBP is considered a fair predictor of MT [20], while the Shock Volume, which assesses the SI iteratively over multiple time points is more promising [21]. As demonstrated in previous studies [22,23], patients who receive more than 1000 mL of fluids in the pre-hospital settings and are “transient” or “no responders” will be more likely to require blood products and will have an increase in mortality.

The GCS 3 on the scene as an independent variable of MT can be read in two ways: patients with severe hemorrhage may not have sufficient cerebral perfusion to maintain a state of consciousness, or alternatively, major traumatic events that result in critical bleeding for multi-district injuries are often associated with severe TBI. Moreover, a severe brain injury can be a trigger of early coagulopathy with worsening of bleeding injuries. Only Prince of Wales score [12] included GCS in the calculation. The GCS was a predictor of transfusion needs also in the Prospective Observational Multicenter Major Trauma (PROMMT) study population [24].

In this study, the administration of TXA correlated with the need for MT. Early administration of an anti-fibrinolytic agent was demonstrated effective in improving the outcome of the traumatized patients with hypotension [25] and its use is a standard of treatment in our protocols. Of note, the Italian population of the study was treated on the scene by doctors which were capable in most of cases to diagnose clinically the presence of a critical bleeding and to start the early use of this drug.

In our study penetrating trauma was not recognized as independent predictor of MT. This was different from other scores, such as ABC [14] and Schreiber [15]. In the European experience, many injuries of torso classified as penetrating are superficial and not life threatening. Similarly, the TASH score, derived from TR-DGU which was used also for the validation of our score, does not include penetrating trauma [11].

In hemorrhagic trauma patients, the chest, abdomen, pelvis and extremities are common sites of injuries that cause blood loss. While limb amputation is of immediate clinical evidence and subject to temporary control by tourniquet, the chest-abdomen-pelvic hemorrhages, defined as “non-compressible”, can be addressed by chest and pelvic plain films and by E-FAST. The association between persistent hemodynamic instability in ED despite resuscitation and an anatomical bleeding injury has found to be independent predictor of MT with multivariate analysis. Notably, positive E-FAST was not significantly associated with MT at univariate analysis: a solid organ injury with stable hemodynamics does not need MT and can be often treated with non-operative management [26,27,28]. On the other hand, positive E-FAST became significant in logistic regression of multivariate analysis, when considered together with unstable hemodynamic data. Similarly, the TASH, ABC, Prince of Wales, TBSS, scores demonstrated a predictive value of positive E-FAST and pelvic fracture [11,12,14,18,19].

In civilian setting, major exanguinating limb trauma are infrequent, and to our knowledge the available literature only discusses the usefulness of tournquet, but not the relationship between the presence of one or more amputated limbs and massive transfusion.

Evidences coming from military setting demonstrated a correlation between limb amputation and massive transfusion. In a recent study, Shackelford et al. [29] cohort of US military combat casualties, demonstrated that the presence of two or more amputated limbs below the knee/elbow or of 1 amputated limb abow the knee/elbow was significantly related to the blood component requirement, and that early blood transfusion significantly improved the 24-h and 30-days survival, than delayed transfusion or not transfusion.

In our study, traumatic limb amputation results as an independent predictor of massive transfusion with an OR of 45.066.

This result reinforces the concept that patients suffering from this type of injuries require a prompt and aggressive approach, according with the Damage Control Strategy, combining the principles of Damage Control Orthopedics [30] and those of Damage Control Resuscitation [9].

Furthermore, our study confirms the role of coagulopathy as an important predictor of critical bleeding. Several studies showed a double origin of trauma-induced coagulopathy. One cause of TIC is the dilution of factors and hypothermia induced by emergency infusions. A second cause is the inhibition of thrombin generation induced by the biological response to hypoperfusion through the expression of thrombomodulin [27]. According to Maegele et al. [28] endothelial damage determines a phenomenon of auto-heparinization, while a secondary coagulopathy originates from inadequate resuscitation leading to acidosis, hypothermia and hemodilution. The value of PTT ≥ 40.0 s showed the second (after limb amputation) highest OR among variables predictive of MT.

Finally, we used the TR-DGU because it was built according to the criteria of Utstein [31] with a template that considers the 34 “core” variables common to all the European trauma registers, including that of Niguarda. The validation of Italian data on the German TR-DGU register allowed to assess the reliability of the “Milano Score” which can therefore be a useful and simple tool for urgent decision making for the clinician.

Our study is not without limitations. One limitation of this study is the small number of initial patients who were prospectively included, even if 139 trauma patients with hemodynamic instability consecutively admitted in a single institution represents a reliable population. Another concern may be the criteria used to define critical bleeding. Although largely debated, we chose 4 or more PRBC within one hour to select a severe condition of acute hemorrhage where timely MT activation is essential for survival. The PH hemodynamic variables were not independent predictors of MT, and this may limit the usefulness of the score. It is a common consideration that early hemorrhagic shock can be well tolerated by young and healthy people with only subtle signs (pale, clammy skin, tachycardia, agitation) and hypotension may occur late, once in the hospital, even in the presence of severe bleeding injuries. In a few minutes after admission all the variables of the score can be available driving the activation of MT protocols. Therefore, when long transportation time is expected, most of the score variables are obtainable in prehospital settings with point of care technology (prehospital ultrasound, rapid coagulation test). Other emerging predictors of MT activation, such as rotational thromboelastometry or thromboelastography has not been investigated. However, incorporating more elements into the decision to trigger MT comes with potential drawbacks, including the delay of activation while awaiting laboratory results. Finally, a single dataset for the present analysis has been produced from the TraumaRegister DGU^®^, containing all variables for the calculation of the score and a number of patients had to be excluded due to missing data, with possible bias.

## 5. Conclusions

Unstable hemodynamics in trauma patients, combined with altered coagulation and evidence of a bleeding injury are significantly associated with the need of MT. Massive transfusion plays a pivotal role during the damage control resuscitation, and an early and effective administration of blood components in a predefined ratio improves patient survival.

The proposed 9 variable easy-to use-score obtained during the early care of trauma patient can be used to predict critical bleeding, allowing an early administration of MT, helping to effectively reduce acute traumatic coagulopathy.

## Figures and Tables

**Table 1:** ***Left:*** Demographic and hemodynamic variables of patients from Milano trauma registry. ***Right:*** Variables significantly associated with MT at univariate analysis.

Variable	MT− (N° = 78)	MT+ (N° = 61)	*p*-Value	Variable	Odds Ratio C.I.95%
Age (M ± SD) (median)	45 (±20) 41	47 (±17) 47	0.35	-	-
Gender Male (N° %)	60 (76.9%)	42 (68.9%)	0.08	-	-
ISS (M ± SD) (median)	33 (±14) 33	44 (±19) 43	<0.001	-	-
Penetrating (%)	5 6.4	8 13.1	0.18	Penetrating trauma	2.20 0.68–7.11
SBP PH, mmHg (M ± SD) (median)	83.2 (±20.9) 80.00	79.7 (±31.2) 80.00	0.44	-	-
HR PH, bpm (M ± SD) (median)	94.8 (±29.3) 98.00	106.3 (±37.2) 10.5	0.048	HR PH ≥ 100	2.96 1.43–6.10
GCS PH (M ± SD) (median)	11.31 (±4.5) 14	9.57 (±5.15) 10.50	0.040	GCS PH 3	3.11 1.23–7.86
RTS PH (M ± SD) (median)	5.955 (±1.64) 6.38	5.138 (±2.38) 6.17	0.489	-	-
SBP ED, mmHg (M ± SD) (median)	82.95 (±21) 83.00	74.07 (±21.09) 117.50	0.017	SBP SR ≤ 70	2.03 1.13–4.68
HR ED (M ± SD) (median)	96.15 (±27.87) 95.00	110.2 (±30.3) 77.00	0.006	HR SR ≥ 120	3.95 1.79–8.67
GCS ED (M ± SD) (median)	7.04 (±5.6) 3.0	5.81 (±4.9) 3.0	0.187	-	-
TXA PH N° (%)	12 (15.4)	19 (31.1)	0.03	TXA PH	2.48 1.09–5.64

ED Emergency Department, GCS Glasgow Coma Score, HR Heart Rate, ISS Injury Severity Score, M mean, MT Massive Transfusion, N° number, RTS Revised Trauma Score, SBP Systolic Blood Pressure, SD standard deviation, TXA Tranexamic Acid.

**Table 2:** ***Left:*** Laboratory parameters and types of injuries in patients of Milano Trauma registry. ***Right:*** variables significantly associated with MT at univariate analysis.

Variable	MT−	MT+	*p* Value	Variable	Odds Ratio C.I.95%
pH (M ± SD) (median)	7.27 (±0.11) 7.30	7.20 (±0) 7.26	0.48	-	-
Lactates mmol/L(M ± SD) (median)	3.58 (±2.58) 2.98	5.87 (±3.90) 4.30	<0.001	-	-
BE mmol/L(M ± SD) (median)	−5.36 (±4.54) −5.60	−9.57 (±5.77) −8.40	<0.001	BE ≤ 9 mmol/L	5.06 2.28–11.24
Hb gr/dL(M ± SD) (median)	11.66 (±2.24) 11.60	9.55 (±2.20) 9.50	0.895	Hb ≤ 10 g/dL	4.37 2.06–9.31
PLT × 10^9^ (M ± SD) (median)	218.3 (±67.25) 216.00	190.46 (±73.94) 183.00	0.028	PLT < 150 × 10^9^	2.84 1.19–6.82
INR (M ± SD) (median)	1.33 (±0.39) 1.24	1.66 (±0.62) 1.58	< 0.001	INR > 1.50	8.44 2.85–24.98
PTT sec (M ± SD) (median)	33.51 (±16.92) 30.10	53.01 (±31.26) 43.90	<0.001	PTT ≥ 40.0 s	11.16 4.30–28.99
Pelvis fracture N° 68	31 (45.6%)	37 (54.4%)	0.01	-	2.13 1.08–4.24
Femur fracture N° 39	21 (53.9%)	18 (46.1%)	0.70	-	0.99 0.48–2.08
Limb amputation N° 10	1 (10%)	9 (90%)	0.02	-	13.33 1.64–108.36
Abd E-FAST pos N° 59	31 (52.5%)	28 (47.5%)	0.42	-	1.67 0.85–3.29
Haemothorax N° 20	7 (35%)	13 (65%)	0.03	-	3.31 1.25–8.73

BE Base Excess, E-FAST Extended Foscused Abdominal Sonography for Trauma, Hb Haemoglobin, INR International Normalized Ratio, PLT platelet, PTT Partial Thromboplastin Time.

**Table 3:** Independent predictors of MT with multivariate analysis from Milano Trauma Registry.

Variables	*p*-Value	Odds-Ratio	C.I. 95% Lower	C.I. 95% Upper	Score
GCS 3 PH	0.061	3.925	0.939	16.409	1
TXA PH	0.045	4.059	1.030	16.002	1
Penetrating trauma	0.040	7.209	1.089	47.709	1
Hemothorax	0.077	3.303	0.879	12.409	1
Pelvic fracture	0.109	2.722	0.800	9.260	1
E-FAST pos	0.021	3.747	1.225	11.467	1
HR ED ≥ 120 bpm	0.008	4.304	1.457	12.711	1
SBP ED ≤ 70 mmHg	0.028	3.569	1.151	11.064	1
PTT ≥ 40.0 s	<0.001	13.616	3.782	49.022	2
					*p*.max: 10
Limb amputation	0.001	45.066	4.297	472.628	

ED Emergency Department, E-FAST Extended Focused Sonography for Trauma, GCS Glasgow Coma Score, HR Heart rate, PTT Partial Thromboplastin Time, SBP Systolic Blood Pressure, TXA Tranexamic Acid.

**Table 4:** Demographic variables of 905 patients from TR-DGU with at least one episode of systolic hypotension.

Variable	MT − (N° 635)	MT + (N° 270)	*p* Value
Age (M ± SD) (median)	54.5 (21.4) 55	51.1 (20.7) 51	0.82
Gender M (N°, %)	444 (69.9%)	186 (68.9%)	0.78
ISS (M ± SD) (median)	29.7 (14.7) 29	40.0 (15.8) 41	<0.001
Mortality (N°, %)	180 (28.3%)	123 (45.6%)	<0.001

ISS Injury Severity Score, M mean, SD Standard Deviation.
